# A polyphenol fraction from *Rosa multiflora* var. platyphylala reduces body fat in overweight humans through appetite suppression – a randomized, double-blind, placebo-controlled trial

**DOI:** 10.1186/s12906-024-04487-1

**Published:** 2024-05-21

**Authors:** Heggar Venkataramana Sudeep, Puwar Prithviraj, Thomas V Jestin, Kodimule Shyamprasad

**Affiliations:** 1 Department of Biomedicinal Research, R&D Center for Excellence, Vidya Herbs Pvt Ltd, No. 14/A, KIADB, Jigani Industrial Area, Anekal Taluk, Bangalore, Karnataka 560105 India; 2 Anand Multispeciality Hospital, Gorwa, Vadodara, Gujarat 390016 India; 3 Leads Clinical Research and Bioservices Pvt Ltd, Bangalore, India

**Keywords:** Rose petal, Polyphenols, Body fat, Appetite

## Abstract

**Background:**

Rosa species are rich sources of polyphenols with physiological functions. In this study a polyphenol-rich *Rosa multiflora* (var. platyphylala) petal extract (RoseFit™) was investigated for weight loss in humans.

**Methods:**

In a randomized, placebo-controlled, parallel-group, double-blind clinical trial seventy overweight male and female subjects (20–50 years) with body mass index (BMI) 25–30 kg/m^2^ were randomly allocated to the active treatment group (RoseFit) and placebo group in a 1:1 ratio. The subjects received 300 mg capsules twice daily for 12 weeks. The primary efficacy outcome measures included body weight, BMI, and body composition, as determined using Dual-energy X-ray absorptiometry (DEXA). Secondary measures consisted of serum lipid profile and appetite marker (leptin and ghrelin) analyses. Safety analyses included biochemical and hematological assessments.

**Results:**

At the end of the study, a marked reduction in body weight (-1.20 ± 2.62 kg, *p* < 0.05) and BMI from baseline was observed in the RoseFit group. In addition, the body fat % (RoseFit = -1.69 ± 2.59%, placebo = 0.96 ± 3.21%; *p* < 0.001) and fat mass (RoseFit = -1.75 ± 1.80 kg, placebo = 1.61 ± 3.82 kg; *p* < 0.001) were significantly abated in RoseFit group. Importantly, the lean mass was maintained during the intervention. RoseFit ingestion significantly increased the serum leptin levels compared to the placebo (4.85%; *p* < 0.05). Further, RoseFit group showed reduction in the hunger hormone ghrelin level (2.27%; *p* < 0.001) from baseline to the end of study, compared to the placebo. The subjective evaluation of appetite using visual analog scale (VAS) questionnaires further confirmed the appetite-suppression effects of RoseFit. The lipid profile significantly improved in RoseFit-treated subjects. No serious adverse events were observed during the study, indicating the tolerability of RoseFit.

**Conclusions:**

Supplementation with RoseFit significantly impacts body weight management and can thus be a potential nutraceutical ingredient for sustainable weight loss.

**Trial registration:**

CTRI/2019/10/021584 dated 09/10/2019

**Supplementary Information:**

The online version contains supplementary material available at 10.1186/s12906-024-04487-1.

## Background

The world population is vulnerable to serious health concerns owing to its modern-day lifestyle, stress, and food habits. Over the years, obesity has contributed significantly to health complications, such as type 2 diabetes, cardiovascular diseases, fatty liver disease, and various types of cancer [1, 2]. The etiology of obesity includes genetic inheritance, metabolism, and environmental and economic factors. Obesity is a multifactorial disease, the most common attribute of which is hedonic eating, associated with minimal physical activity [3]. Weight management includes behavioural and lifestyle changes, healthy eating habits besides the medications to regulate appetite, fat metabolism, and surgical interventions [4]. Anti-obesity medications are associated with undesirable side effects. For example, the appetite suppressant drug phentermine is associated with insomnia, increased blood pressure, and dry mouth [5]. Orlistat, a drug that slows intestinal fat absorption via lipase inhibition, has gastrointestinal side effects [6]. Natural products have gained considerable attention in the global market as functional ingredients for managing obesity, with fewer side effects [7]. Various phytochemicals, unsaturated fatty acids, and fibers present in herbal products contribute significantly to weight loss and other health benefits [8].

The genus Rosa comprises approximately 200 species with different varieties of flowering plants. Distributed mostly in sub-tropical regions, Rosa species are cultivated as ornamentals. Many rose varieties have medicinal properties. *Rosa multiflora* Thunb. is a perennial plant with reported pharmacological effects such as antioxidant, antimicrobial, anti-inflammatory, and skin-whitening properties [9, 10]. Liaudanskas et al. quantitatively analyzed fruit extracts from different *Rosa* L. cultivars, including *R. multiflora* for polyphenol and flavonoid contents and reported their antioxidant activity [11]. More recently, Park et al. studied the anti-inflammatory activity of solvent-extracted fractions of *R. multiflora* flowers using Raw 264.7 cells [12]. Except for some scientific literature, there is a lack of research on the various pharmacological benefits of *R. multiflora* preparations. Previously, we reported the anti-obesity activity of a standardized *R. multiflora* petal extract (RoseFit™) in 3T3-L1 adipocytes, and a high-fat diet (HFD)-induced murine model [13]. To validate the therapeutic claims, we conducted a clinical trial on overweight human subjects and reported whether the ingestion of RoseFit alters body composition, lipid profile, and appetite in individuals enduring weight loss effects.

## Methods

### Investigational product

Powdered rose petal extract (RoseFit^™^) from *R. multiflora* var. platyphylala, standardized to contain 2–3% isoquercetin and 63.82% of total polyphenol content, was used in the present study. The extract was formulated with maltodextrin in the form of a capsule, so that each 300 mg capsule contained 200 mg of RoseFit. The placebo capsules contained equivalent weight of maltodextrin and were identical to the extract capsules in colour and appearance.

### Ethical approval

The study protocol was approved by the Institutional Ethics Committee of the Anand Multispecialty Hospital, Vadodara, Gujarat, India (LCBS-VH-47 dated 27/08/2019). The trial was prospectively registered in the Clinical Trial Registry, India (CTRI/2019/10/021584 dated 09/10/2019). This study was conducted according to the International Conference on Harmonization of Technical Requirements for Registration of Pharmaceuticals for Human Use –Good Clinical Practice (ICH-GCP) guidelines. The study protocol adhered to the Declaration of Helsinki guidelines.

### Subjects

Seventy healthy male and female subjects aged 20–50 years, with a BMI of 25–30 kg/m^2^ were enrolled in the trial. The subjects were detailed regarding the study objective, protocol, and possible risk factors involved in the study. All participants signed a written informed consent form before the initiation of the study. The subject enrolment was based on the following exclusion criteria: (1) subjects who had experienced unexplained weight loss or gain six months prior to screening; (2) pathophysiological or genetic syndrome associated with obesity (Cushing’s syndrome, Turner’s syndrome, Prader-Willli syndrome); (3) intake of over-the-counter weight loss agents, centrally acting appetite suppressants in the prior six months of screening; (4) subjects who suffered from any chronic health conditions (e.g., diabetes, hypertension, chronic renal failure, heart, thyroid, and liver disease); (5) subjects who were allergic to herbal products including the constituents of the investigational product; (6) history of chronic metabolic disease, psychiatric illness, drug abuse, smoking, addiction to alcohol, bariatric surgery, cardiac surgery, or endocrine abnormalities; (7) participation in any other clinical trial during the past three months of screening; (8) any additional conditions according to the investigator would warrant exclusion from the study.

### Study design

The present clinical trial was conducted at the Anand Multispecialty Hospital in Vadodara, Gujarat. This study was a randomized, double-blind, placebo-controlled, parallel, two-arm trial. Overweight subjects meeting all the inclusion criteria and no exclusion criteria were enrolled during the screening visit. The participants were randomly assigned to the placebo and test (RoseFit) groups at a 1:1 ratio (35 subjects allocated to each group). Block randomization was used for the subject allocation. Briefly, the interventions were assigned a code, and a random allocation sequence was generated with random block size (R statistical software). The random blocks of 8, 16, 12 and 10 were used. The randomization sequence was controlled by the blinded statistician. All the subjects were enrolled by the investigator. The participants and investigators were blinded to the interventions with the use of unique identification code in the IP labels.

All the subjects were instructed to take 300 mg capsules twice daily (30 min before breakfast and dinner) for 12 weeks. The dosage of 400 mg of RoseFit per day (200 mg in each capsule; twice daily) was established based on the pharmacologically active dose (PAD) calculation by body surface area conversion factor (BSA-CF) method [14].

**Fig. 1:**
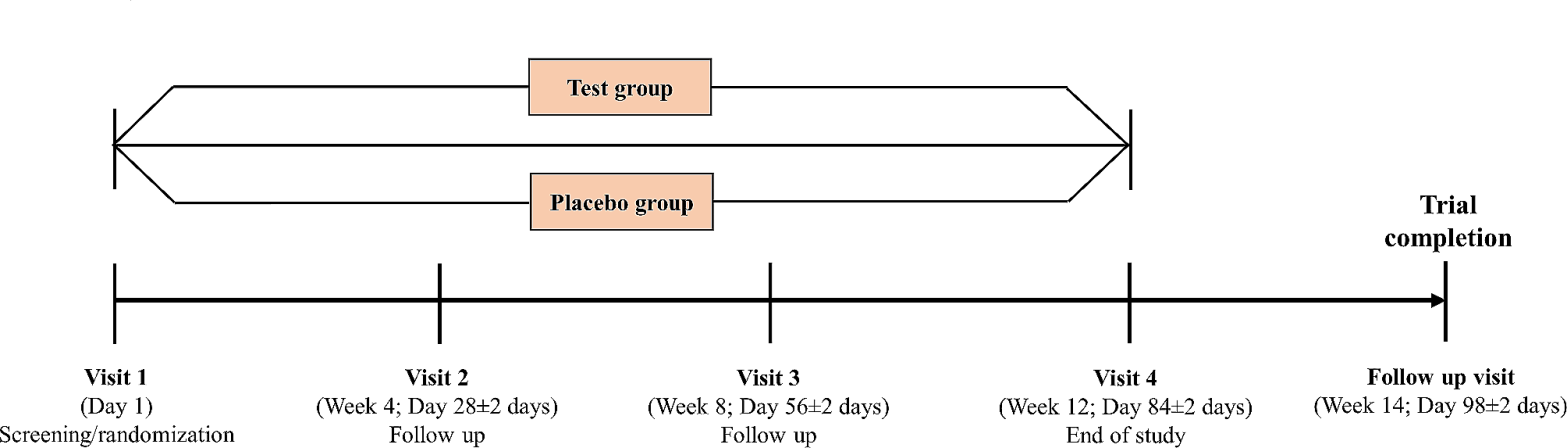
Study design and visit schedule

The subjects’ diets were not controlled during the study. However, the participants were advised to follow a common diet (2000–2500 cal). A diet chart with caloric information was provided to the participants (Supplementary file 1). The details of the study visits are shown in Fig. 1.

### Sample size determination

The sample size was calculated by considering the body weight changes between the groups to be clinically significant. Assuming a common standard deviation of 2.78 at the end of treatment, 31 per group would be sufficient to detect a difference of 1.45 in mean difference b/w the two treatments with power of 80% and a 0.05. 2-sided level of significance. A detailed calculation of the sample size is provided in Supplementary file 2.

### Study outcomes

Primary outcome measures included body weight, BMI, and changes in body composition. Body composition was analyzed using dual-energy X-ray absorptiometry (DEXA; Lunar iDXA, GE Healthcare, India). The secondary outcomes were changes in appetite markers (leptin and ghrelin) and lipid profiles (total cholesterol (TC), triglycerides (TG), low-density lipoprotein (LDL)-cholesterol, high-density lipoprotein (HDL)-cholesterol, TC to HDL-cholesterol ratio, and LDL-cholesterol to HDL-cholesterol ratio). The appetite markers leptin and ghrelin were analyzed using commercial ELISA (Enzyme-linked immunosorbent assay) kits following the manufacturer’s instructions. Furthermore, appetite was assessed using a visual analog scale (VAS) questionnaire [15]. Health-related quality of life (HRQL) was assessed using the validated 12-item short form health Survey (SF-12) questionnaire, which covers physical and mental health aspects [16, 17].

The safety evaluation included observing adverse (AE) and severe adverse events (SAE) and blood biochemical parameters such as aspartate aminotransferase (AST), alanine aminotransferase (ALT), and alkaline phosphatase (ALP) for liver function, serum creatinine for renal function, and hematological parameters.

### Statistical analysis

The data were analyzed using R-statistical software (version 4.2.1) and presented as mean ± standard deviation (SD). Changes within the group from baseline to the end of treatment were analyzed using a paired t-test. Differences between the groups were analyzed using an independent t-test. Data were considered statistically significant at *p* < 0.05.

## Results

### Demographic characteristics of subjects

**Fig. 2:**
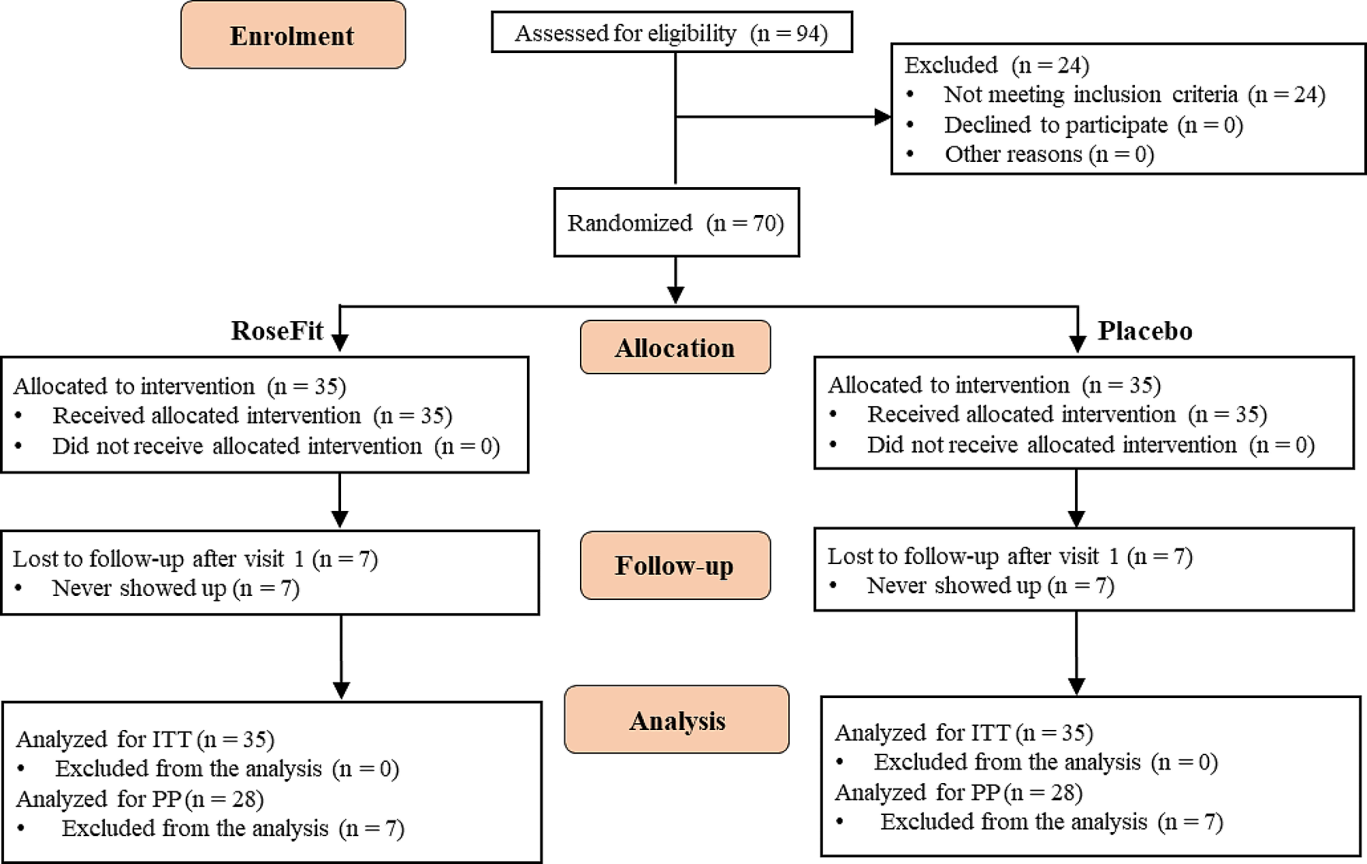
Study participant flow diagram

Subject enrolment was initiated on 17th June 17, 2020, and completed on 26th March 26, 2021. Figure 2 shows the flow diagram of the enrolled subjects. Overall, 94 volunteers were screened for enrolment, of which 70 subjects meeting the eligibility criteria were randomly allocated to the test and placebo groups and received RoseFit and placebo capsules, respectively. Seven subjects each from the RoseFit and placebo groups were lost to follow-up. These subjects never showed up after the first visit. Therefore, 28 participants from each group completed the trial. The efficacy parameters were measured using Intention-to-treat (ITT) and per-protocol (PP) analyses. PP analysis is provided in Supplementary file 3.

Table 1 shows the demographic characteristics of the participants at the baseline. Baseline characteristics such as age, sex, height, and weight of the subjects did not vary significantly between the groups.

**Table 1:** Baseline demographic characteristics of subjects

Variable	RoseFit (*n* = 35)	Placebo (*n* = 35)	*p*-value
Age	34.71 ± 7.78	34.14 ± 7.65	0.758^1^
Gender (M/F)	13/22	12/23	0.803^2^
Height (cm)	159.43 ± 8.02	158.37 ± 7.56	0.572^1^
Weight (kg)	71.49 ± 8.67	69.66 ± 7.81	0.358^1^

Values are mean ± SD, and data were statistically analyzed using^1^ independent sample t-test and ^2^ Chi-square test. M/F; Male/Female.

### Effect of RoseFit on body weight and BMI

Table 2 shows the effect of RoseFit administration on body weight and BMI of the subjects. The mean body weight in the RoseFit group was significantly reduced from 71.71 ± 10.56 kg at baseline to 70.51 ± 10.11 kg after 12 weeks (*p =* 0.011) whereas the placebo group showed an increase in the body weight at the end of treatment (*p =* 0.011). The mean changes in body weight were significant between the groups (*p* < 0.001). A similar trend was observed in the BMI data. The mean BMI was abated in RoseFit group from baseline value of 28.03 ± 1.50 kg/m^2^ to 26.82 ± 1.44 kg/m^2^ at the end of study (*p* < 0.001). In contrast, the BMI increased from baseline (27.71 ± 1.54 kg/m^2^) to the end of the study (28.39 ± 1.52 kg/m^2^) in the placebo group (*p* < 0.001). The change in BMI from the baseline was significant between the treatment groups (*p* < 0.001).

**Table 2:** Effect of RoseFit on body weight and BMI of subjects

Parameter	RoseFit (*N* = 35)	Placebo (*N* = 35)	*p*-value (between groups)
**Body weight (kg)**			
Baseline	71.71 ± 10.56	68.28 ± 11.20	0.193†
Visit 4 (12 weeks)	70.51 ± 10.11	70.57 ± 9.83	0.979†
Change	-1.20 ± 2.62	2.29 ± 5.03	< 0.001†***
*p*-value (Baseline vs. Visit 4)	0.011#*	0.011#*	
**BMI (kg/m**^**2**^)			
Baseline	28.03 ± 1.50	27.71 ± 1.54	0.373†
Visit 4 (12 weeks)	26.82 ± 1.44	28.39 ± 1.52	< 0.001†***
Change	-1.21 ± 0.89	0.69 ± 0.70	< 0.001†***
*p*-value (Baseline vs. Visit 4)	< 0.001#***	< 0.001#***	

Values are presented as mean ± SD. Change = Visit 4 – Baseline

#Paired t-test; †Independent t-test; *p < 0.05, ***p < 0.001

### Effect of RoseFit on body composition

Table 3 shows the body composition analysis of the subjects. A 12-week ingestion of RoseFit significantly reduced the body fat from 46.53 ± 7.02% to 44.84 ± 7.84% (*p* < 0.001). In contrast, there was no significant change in the body fat % of the placebo group from baseline to the end of study (*p* = 0.084). The change in body fat % from baseline to the end of treatment was significant (*p* < 0.001) between the RoseFit (-1.69 ± 2.59%) and placebo (0.96 ± 3.21%) groups.

**Table 3:** Effect of RoseFit on the body composition of subjects (DEXA analysis)

Parameter	RoseFit (*N* = 35)	Placebo (*N* = 35)	*p*-value (between groups)
**Body fat (%)**			
Baseline	46.53 ± 7.02	46.92 ± 8.78	0.839†
Visit 4 (12 weeks)	44.84 ± 7.84	47.88 ± 9.03	0.137†
Change	-1.69 ± 2.59	0.96 ± 3.21	< 0.001†***
*p*-value (Baseline vs. Visit 4)	< 0.001#***	0.084#	
**Fat mass (kg)**			
Baseline	32.11 ± 5.73	31.03 ± 8.13	0.521†
Visit 4 (12 weeks)	30.36 ± 6.17	32.64 ± 7.62	< 0.174†
Change	-1.75 ± 1.80	1.61 ± 3.82	< 0.001†***
*p*-value (Baseline vs. Visit 4)	< 0.001#***	0.017#*	
**Lean mass (kg)**			
Baseline	37.24 ± 8.28	34.75 ± 7.81	0.201†
Visit 4 (12 weeks)	37.79 ± 8.07	35.43 ± 7.87	0.219†
Change	0.55 ± 2.03	0.68 ± 2.12	0.806†
*p*-value (Baseline vs. Visit 4)	0.116#	0.077#	

Values are presented as mean ± SD. Change = Visit 4 – Baseline

#Paired t-test; †Independent t-test; *p < 0.05, ***p < 0.001

Further, RoseFit treatment significantly reduced the fat mass from 32.11 ± 5.73 kg at baseline to 30.36 ± 6.17 kg (*p* < 0.001) after 12 weeks whereas in the placebo group the mean fat mass was increased to 32.64 ± 7.62 kg from a baseline measure of 31.03 ± 8.13 kg (*p* = 0.017). The mean reduction of fat mass in the RoseFit group (-1.75 ± 1.80 kg, *p* < 0.001) was significant compared to the placebo (1.61 ± 3.82 kg). Interestingly, both the RoseFit and placebo groups showed an increasing trend in lean mass from the baseline to the end of treatment. However, these changes were not statistically significant.

The body weight, BMI and the body composition parameters were analyzed using ANCOVA with baseline as co-variate (Table 4).

**Table 4:** Analysis of covariance of body weight, BMI and body composition parameters (Per-protocol analysis)

Parameter	Visit	RoseFit		Placebo		*p*-value (between groups)
		*N*		*N*		
Body weight (kg)	Baseline	28	72.75 ± 8.61	28	68.98 ± 7.46	< 0.001†**
	Visit 4	28	69.27 ± 8.61	28	70.93 ± 7.36	
BMI	Baseline	28	28.18 ± 1.53	28	27.86 ± 1.56	< 0.001†**
	Visit 4	28	26.80 ± 1.54	28	28.65 ± 1.48	
Body fat %	Baseline	28	45.88 ± 7.06	28	47.87 ± 8.43	< 0.001†**
	Visit 4	28	43.77 ± 7.84	28	49.07 ± 8.61	
Fat mass (kg)	Baseline	28	31.97 ± 5.89	28	31.57 ± 8.41	< 0.001†**
	Visit 4	28	29.78 ± 6.29	28	33.59 ± 7.57	
Lean mass (kg)	Baseline	28	37.96 ± 8.23	28	34.04 ± 7.50	0.915†
	Visit 4	28	38.66 ± 7.89	28	34.89 ± 7.65	

†ANOVA with baseline data as co-variate

**p < 0.001

### Effect of RoseFit on the appetite regulation

**Fig. 3:**
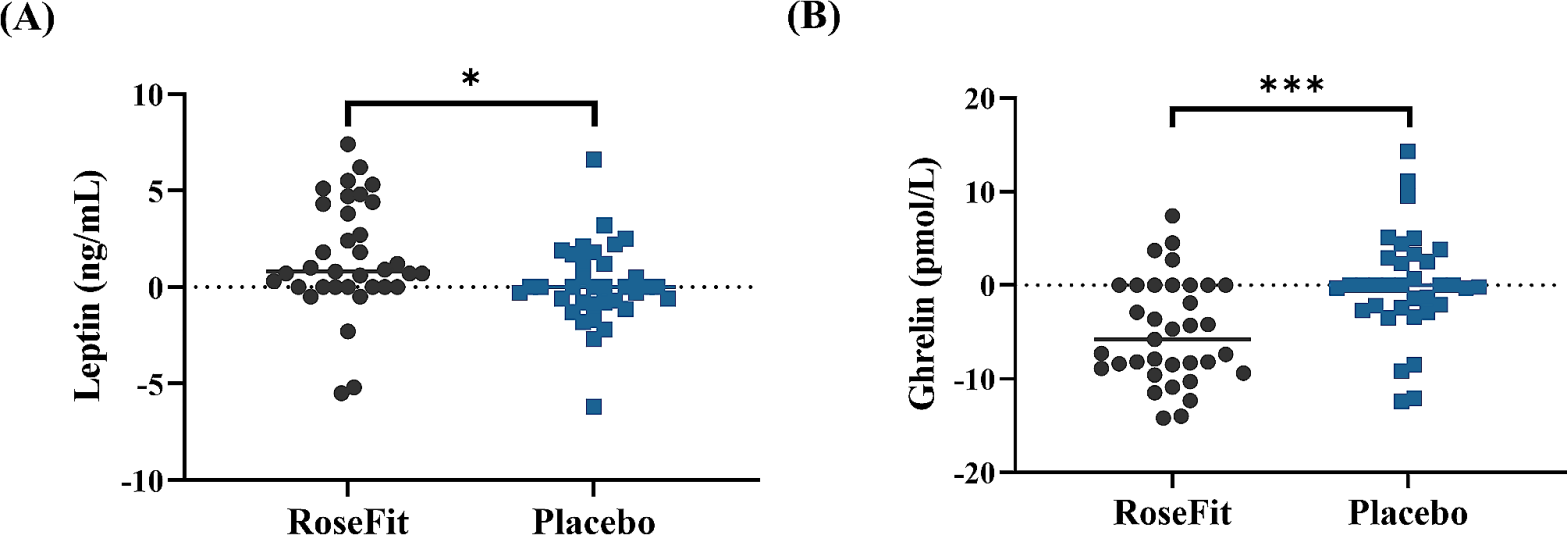
Effect of RoseFit on appetite regulating hormones. Serum levels of leptin (**A**) and ghrelin (**B**) **a**nalyzed at the end of study. The data were analysed using independent t test. *p < 0.05 and ***p < 0.001; ns: not significant

In the present study, serum levels of appetite biomarkers were assessed using ELISA (Fig. 3). The increase in leptin hormone level from baseline to the end of treatment was 1.52 ± 2.88 ng/mL and 0.10 ± 2.07 ng/mL in RoseFit and placebo groups respectively. The change in serum leptin level in the RoseFit group was significant compared to that in the placebo group (*p* < 0.05, Fig. 3A). There was a marked reduction in the ghrelin level of the RoseFit group (-4.98 ± 5.49 pmol/L) from baseline, whereas no change in the hormone level was noticed in the placebo group (0.00 ± 5.56 pmol/L). These changes were significant between the groups (*p* < 0.001, Fig. 3B).

The use of a VAS questionnaire is a promising and reasonable approach for ascertaining appetite and satiety [18]. Table 5 shows a summary of VAS scores. The RoseFit group showed a significant reduction (*p* < 0.001) in the hunger score from baseline (89.43 ± 7.65) to the end of treatment (72.57 ± 13.58), while there was only a marginal change in the placebo group. The change in the hunger score of the RoseFit group was significant compared to that of the placebo group (*p* < 0.001). Further, the satiety and fullness scores were markedly increased in RoseFit group with respective changes of 7.14 ± 9.87 and 8.29 ± 9.23 from baseline (*p* < 0.001). These changes were significant compared to those in the placebo group (*p* < 0.001). Concurrently, the prospective food consumption subsided in the RoseFit group (-8.86 ± 8.67, *p* < 0.001), whereas the food consumption score was insignificantly increased from baseline (2.57 ± 9.50) in the placebo group. The RoseFit group showed a significant difference compared with the placebo group (*p* < 0.01).

**Table 5:** Summary of perceived hunger and satiety scores using Visual Analog Scale (VAS)

Parameter	RoseFit (*N* = 35)	Placebo (*N* = 35)	*p*-value (between groups)
**Hunger score**			
Baseline	89.43 ± 7.65	89.71 ± 8.57	0.883†
Visit 4 (12 weeks)	72.57 ± 13.58	88.57 ± 9.74	< 0.001†***
Change	-16.86 ± 13.23	-1.14 ± 7.18	< 0.001†***
*p*-value (Baseline vs. Visit 4)	< 0.001#***	0.353#	
**Satiety score**			
Baseline	78.57 ± 8.79	79.43 ± 12.35	0.739†
Visit 4 (12 weeks)	85.71 ± 6.08	77.71 ± 9.73	< 0.001†***
Change	7.14 ± 9.87	1.71 ± 7.07	< 0.001†***
*p*-value (Baseline vs. Visit 4)	< 0.001#***	0.160#	
**Fullness score**			
Baseline	73.71 ± 7.70	74.00 ± 9.46	0.890†
Visit 4 (12 weeks)	82.00 ± 7.59	75.14 ± 7.02	< 0.001†***
Change	8.29 ± 9.23	1.14 ± 6.31	< 0.001†***
*p*-value (Baseline vs. Visit 4)	< 0.001#***	0.292#	
**Prospective food consumption**			
Baseline	86.00 ± 7.36	86.29 ± 10.87	0.898†
Visit 4 (12 weeks)	77.14 ± 6.67	83.71 ± 6.90	< 0.001†***
Change	-8.86 ± 8.67	2.57 ± 9.50	0.005†**
*p*-value (Baseline vs. Visit 4)	< 0.001#***	0.119#	

Values are presented as mean ± SD. Change = Visit 4 – Baseline

#Paired t-test (baseline vs. visit 4); †Independent t-test; **p < 0.01, ***p < 0.001

### Effect of RoseFit on serum lipid profile

The serum levels of TC, TG, HDL-cholesterol, and LDL-cholesterol were measured at baseline and after 12 weeks of treatment. As shown in Table 6, the RoseFit group showed a significant reduction in parameters such as TC, TG, LDL-cholesterol, TC/HDL ratio, and LDL/HDL ratio from baseline to the end of the study (*p* < 0.001), whereas HDL-cholesterol increased after 12-week treatment (*p* < 0.01). In contrast, the placebo group showed a significant increase in TC (*p* < 0.01), LDL-cholesterol, and the TC/HDL ratio (*p* < 0.05). The other lipid parameters were not significantly altered in the placebo group. The change from baseline in all parameters except HDL-cholesterol was significantly different in the RoseFit treatment group compared to that in the placebo group.

**Table 6:** Effect of RoseFit on serum lipid profile of subjects

Parameter	RoseFit (*N* = 35)	Placebo (*N* = 35)	*p*-value (between groups)
**Total cholesterol (mg/dL)**			
Baseline	181.82 ± 17.17	185.97 ± 18.27	0.330†
Visit 4 (12 weeks)	175.46 ± 16.65	190.69 ± 20.56	0.0011†**
Change	-6.36 ± 6.81	4.71 ± 8.63	< 0.001†***
*p*-value (Baseline vs. Visit 4)	< 0.001#***	0.003#**	
**Triglycerides (mg/dL)**			
Baseline	176.63 ± 23.31	180.06 ± 21.93	0.528†
Visit 4 (12 weeks)	170.43 ± 25.69	179.69 ± 25.79	0.137†
Change	-6.20 ± 8.27	-0.37 ± 8.45	0.005†**
*p*-value (Baseline vs. Visit 4)	< 0.001#***	0.796#	
**LDL-cholesterol (mg/dL)**			
Baseline	109.46 ± 16.67	112.86 ± 17.16	0.404†
Visit 4 (12 weeks)	102.86 ± 16.76	116.49 ± 18.69	0.002†**
Change	-6.60 ± 7.01	3.63 ± 7.94	< 0.001†***
*p*-value (Baseline vs. Visit 4)	< 0.001#***	0.011#*	
**HDL-cholesterol (mg/dL)**			
Baseline	41.37 ± 5.13	41.26 ± 4.01	0.918†
Visit 4 (12 weeks)	42.20 ± 5.32	41.43 ± 3.88	0.491†
Change	-0.83 ± 1.36	0.17 ± 1.60	0.069†
*p*-value (Baseline vs. Visit 4)	< 0.001#***	0.530#	
**Total cholesterol/HDL-cholesterol ratio**			
Baseline	4.47 ± 0.78	4.55 ± 0.60	0.658†
Visit 4 (12 weeks)	4.23 ± 0.73	4.63 ± 0.60	0.014†
Change	-0.24 ± 0.27	0.09 ± 0.25	< 0.001†***
*p*-value (Baseline vs. Visit 4)	< 0.001#***	0.047#*	
**LDL/HDL ratio**			
Baseline	2.70 ± 0.62	2.76 ± 0.49	0.662†
Visit 4 (12 weeks)	2.49 ± 0.60	2.83 ± 0.51	0.013†
Change	-0.21 ± 0.22	0.07 ± 0.22	< 0.001†***
*p*-value (Baseline vs. Visit 4)	< 0.001#***	0.063#	

Values are presented as mean ± SD. Change = Visit 4 – Baseline

#Paired t-test (baseline vs. visit 4); †Independent t-test; **p < 0.01, ***p < 0.001

### Assessment of the effect of RoseFit on perceived HRQL

The SF-12 is a reliable tool that is widely used to assess self-reported HRQL [19]. Table 7 summarizes the effect of RoseFit on perceived HRQL at the baseline and at the end of the study. The total SF-12 score was increased by 2.46 ± 2.77 from baseline in RoseFit group (*p* < 0.001) whereas the placebo group exhibited marginal variation of -0.71 ± 2.32 from the baseline score. The change in the total score at the end of the 12-week treatment was significant in the RoseFit group compared to the placebo group (*p* < 0.001).

**Table 7:** Summary of health-related quality of life (HRQL) assessment

SF-12 scoring	RoseFit (*N* = 35)	Placebo (*N* = 35)	*p*-value (between groups)
**Total score**			
Baseline	27.91 ± 2.70	27.97 ± 2.81	0.931†
Visit 4 (12 weeks)	30.37 ± 3.64	27.26 ± 3.68	< 0.001†***
Change	2.46 ± 2.77	-0.71 ± 2.32	< 0.001†***
*p*-value (Baseline vs. Visit 4)	< 0.001#***	0.078#	
**PCS-12 score**			
Baseline	11.57 ± 1.04	11.80 ± 0.93	0.336†
Visit 4 (12 weeks)	13.40 ± 1.77	11.49 ± 1.54	< 0.001†***
Change	1.83 ± 1.92	-0.31 ± 1.35	< 0.001†***
*p*-value (Baseline vs. Visit 4)	< 0.001#***	0.176#	
**MCS-12 score**			
Baseline	16.34 ± 2.20	16.17 ± 2.54	0.763†
Visit 4 (12 weeks)	16.97 ± 2.31	15.77 ± 2.68	0.049†*
Change	0.63 ± 1.03	-0.40 ± 1.17	< 0.001†***
*p*-value (Baseline vs. Visit 4)	0.001#**	0.051#	

Values are presented as mean ± SD. Change = Visit 4 – Baseline

#Paired t-test (baseline vs. visit 4); †Independent t-test; *p < 0.05, **p < 0.01, ***p < 0.001

The physical and mental health composite scores (PCS-12 and MCS-12) in the RoseFit group were markedly increased from baseline by 1.83 ± 1.92 and 0.63 ± 1.03, respectively (*p* < 0.001). In the placebo group, the PCS-12 and MCS-12 scores were not significantly reduced from baseline by -0.31 ± 1.35 and − 0.40 ± 1.17 respectively. The observed changes in the PCS-12 and MCS-12 scores were significant in the RoseFit group compared to those in the placebo group.

### Safety assessment of RoseFit

The safety evaluation of RoseFit included liver and renal function tests and hematological measurements. Blood biochemical parameters measured at baseline and at the end of the study were within the normal range. However, there was a significant reduction from baseline in the AST level of the placebo group (*p* < 0.05) and ALT levels of both treatment groups (*p* < 0.001). None of the biochemical parameters changed significantly between the groups (Table 8).

**Table 8:** Safety analysis – Blood biochemical parameters

Parameter	RoseFit (*N* = 35)	Placebo (*N* = 35)	*p*-value (between groups)
**Aspartate aminotransferase (U/L)**			
Baseline	28.60 ± 7.66	31.41 ± 12.18	0.252†
Visit 4 (12 weeks)	28.67 ± 7.17	30.28 ± 11.90	0.497†
Change	0.07 ± 2.46	-1.14 ± 2.62	0.050†
*p*-value (Baseline vs. Visit 4)	0.860#	0.015#*	
**Alanine aminotransferase (U/L)**			
Baseline	32.66 ± 8.62	35.69 ± 12.45	0.241†
Visit 4 (12 weeks)	29.53 ± 8.00	32.81 ± 11.90	0.181†
Change	-3.12 ± 3.85	-2.87 ± 2.71	0.752†
*p*-value (Baseline vs. Visit 4)	< 0.001#***	< 0.001#***	
**Alkaline phosphatase (U/L)**			
Baseline	97.40 ± 12.06	98.73 ± 12.01	0.646†
Visit 4 (12 weeks)	97.96 ± 12.50	97.79 ± 13.06	0.957†
Change	0.55 ± 3.90	0.94 ± 4.09	0.122†
*p*-value (Baseline vs. Visit 4)	0.406#	0.183#	
**Creatinine (mg/dL)**			
Baseline	0.93 ± 0.10	0.92 ± 0.13	0.833†
Visit 4 (12 weeks)	0.93 ± 0.13	0.92 ± 0.17	0.765†
Change	0.01 ± 0.12	0.00 ± 0.11	0.852†
*p*-value (Baseline vs. Visit 4)	0.788#	0.988#	

Values are presented as mean ± SD. Change = Visit 4 – Baseline

#Paired t-test (baseline vs. visit 4); †Independent t-test; *p < 0.05, ***p < 0.001

As shown in Table 9 the hematological parameters of the subjects were normal during the trial and did not change significantly except for a noticeable increase in mean cell hemoglobin from baseline (27.38 ± 1.56) to the end of treatment (27.60 ± 1.39) in the RoseFit group. Furthermore, the vital signs were within normal levels in both groups throughout the study.

**Table 9:** Safety analysis – Hematological parameters

Parameter	RoseFit (*N* = 35)	Placebo (*N* = 35)	*p*-value (between groups)
**Total leukocyte count (mm** ^**3**^ **)**			
Baseline	7837.14 ± 1491.17	7637.14 ± 1439.79	0.570†
Visit 4 (12 weeks)	7857.14 ± 1195.16	7602.86 ± 1289.66	0.395†
Change	20.00 ± 609.15	-34.29 ± 586.09	0.705†
*p*-value (Baseline vs. Visit 4)	0.847#	0.731#	
**Red blood cell count (million/mm**^**3**^)			
Baseline	4.78 ± 0.25	4.85 ± 0.30	0.267†
Visit 4 (12 weeks)	4.78 ± 0.26	4.84 ± 0.29	0.300†
Change	0.00 ± 0.07	0.01 ± 0.08	0.765†
*p*-value (Baseline vs. Visit 4)	0.794#	0.533#	
**Haemoglobin (g/dL)**			
Baseline	13.07 ± 0.69	13.29 ± 0.89	0.253†
Visit 4 (12 weeks)	13.07 ± 0.73	13.29 ± 0.90	0.265†
Change	0.01 ± 0.17	0.01 ± 0.16	1.000†
*p*-value (Baseline vs. Visit 4)	0.845#	0.837#	
**Haematocrit (%)**			
Baseline	40.36 ± 1.69	41.00 ± 2.56	0.222†
Visit 4 (12 weeks)	40.14 ± 2.08	41.09 ± 2.99	0.128†
Change	-0.21 ± 0.95	0.09 ± 1.07	0.206†
*p*-value (Baseline vs. Visit 4)	0.192#	0.605#	
**Mean cell volume (fL)**			
Baseline	84.55 ± 4.17	84.70 ± 5.69	0.903†
Visit 4 (12 weeks)	84.87 ± 4.50	84.60 ± 5.65	0.825†
Change	0.32 ± 1.58	0.10 ± 1.46	0.255†
*p*-value (Baseline vs. Visit 4)	0.242#	0.688#	
**Mean cell haemoglobin (pg)**			
Baseline	27.38 ± 1.56	29.23 ± 12.37	0.388†
Visit 4 (12 weeks)	27.60 ± 1.39	29.33 ± 12.38	0.417†
Change	0.22 ± 0.62	0.11 ± 0.52	0.405†
*p*-value (Baseline vs. Visit 4)	0.043#*	0.236#	
**MCH concentration (%)**			
Baseline	32.41 ± 1.40	32.42 ± 1.34	0.972†
Visit 4 (12 weeks)	32.49 ± 1.58	32.67 ± 1.56	0.633†
Change	0.09 ± 0.87	0.25 ± 0.77	0.394†
*p*-value (Baseline vs. Visit 4)	0.563#	0.059#	
**Platelet count (mm**^**3**^)			
Baseline	272942.90 ± 47711.72	303257.10 ± 57495.44	0.019†*
Visit 4 (12 weeks)	268171.40 ± 67321.65	299314.30 ± 72264.54	0.067†
Change	4771.43 ± 40547.06	3942.86 ± 53040.46	0.942†
*p*-value (Baseline vs. Visit 4)	0.491#	0.663#	

Values are presented as mean ± SD. Change = Visit 4 – Baseline

#Paired t-test (baseline vs. visit 4); †Independent t-test; *p < 0.05

During the study, a total of 15 AEs were reported in the subjects. Three subjects from the RoseFit group and one from the placebo group experienced headache. Other AEs included, stomachache and viral fever experienced by two subjects each from placebo and RoseFit groups, respectively. One subject each in RoseFit and placebo group experienced cold. In addition, one subject in the placebo group reported sore throat, fever, bloating, heartburn and body ache. The details of AEs recorded during the study are given in Supplementary file 4. No serious adverse events were observed during the study. All the AEs reported by the subjects were mild in severity and the causality of the AEs was diagnosed by the investigator as not related to the investigational product. The safety data clearly indicated that daily ingestion of RoseFit for 12 weeks did not cause any side effects.

## Discussion

In this randomized, double-blind trial, we examined the weight-loss potential of RoseFit, a standardized *R. multiflora* petal extract. Daily oral ingestion of 400 mg/day RoseFit for 12 weeks markedly reduced body weight (1.67%) and BMI in overweight individuals. These data were significant compared with those of the placebo group. Several studies involving interventions with polyphenol-rich plant extracts or food have reported significant body weight reduction and increased energy expenditure in individuals [20, 21]. Meta-analysis data from randomized clinical trials (RCTs) strongly suggest the positive effects of polyphenols on body weight [22, 23]. In a long-term study, Guo et al. followed up with over 500 elderly participants for five years, to establish an inverse relationship between total polyphenol intake and body weight and other obesity parameters [24]. In the present study, over 60% of the polyphenols in RoseFit could have substantially contributed to its weight loss effects.

In addition to changes in body weight and BMI, it is important to focus on changes in body composition during a weight loss program [25]. DEXA analysis revealed that concurrent with weight loss, the RoseFit group had a reduced body fat % and fat mass at the end of treatment. These changes were significant compared with those in the placebo group. Importantly, lean mass was maintained without significant alterations following RoseFit administration compared to the placebo group. A reduction in lean body mass is often associated with anti-obesity interventions, which can lead to negative health consequences. Therefore, it is encouraging to observe body fat reduction in the RoseFit group without a significant change in lean mass.

The RoseFit-mediated regulation of appetite is another interesting aspect of this study. It was observed that RoseFit consumption for 12 weeks significantly increased the level of anorexigenic leptin hormone while abating the ghrelin level in subjects compared to the placebo group. Several studies have reported the appetite-suppressing ability of plant polyphenols [26, 27]. Furthermore, the VAS appetite scale, including hunger, satiety, and fullness scores, suggested the involvement of the appetite suppression mechanism of RoseFit polyphenols in improving the body weight of subjects [15].

RoseFit treatment significantly improved the lipid profile of the subjects compared to placebo. In our previously published study, we demonstrated that RoseFit markedly inhibits pancreatic lipase in vitro. In addition, the extract significantly modulated adipogenic protein expression. These mechanisms might be involved in the observed antihyperlipidemic effect of RoseFit in the present study [13].

The impact of RoseFit ingestion on QoL was assessed using SF-12, a 12-item questionnaire derived from SF-36. The SF-12 includes mental and physical component scores, and higher summary scores indicate improved QoL [28]. The RoseFit group showed significantly higher scores at the end of the study than the placebo group. Additionally, blood biochemical and hematological assessments revealed that RoseFit ingestion did not cause any safety concerns during the trial. Interestingly,

To the best of our knowledge, this is the first clinical study to report the weight loss potential of *R. multiflora* petal extract. However, this study has some limitations. Firstly, the study participants were strictly not the representative of obesity and hence the results cannot be extrapolated. However, it can be justified from the study outcome that Porelis can be a promising ingredient controlling the weight gain in overweight individuals, thus preventing the occurrence of obesity. Secondly, the calorie expenditure by exercise and the calorie intake were not statistically measured during the study. Lastly, we did not investigate the effect of RoseFit on the fat distribution. In addition, there were a considerable number of dropouts in this study. This could be due to the lack of motivation and psychological preparation. Lastly, Nonetheless, the main strength of this trial was that we measured body composition using DEXA, along with body weight changes, to clearly understand the impact of RoseFit treatment on fat/lean mass.

## Conclusion

In conclusion, 12-week oral treatment with RoseFit was well tolerated and effective in overweight subjects. RoseFit markedly reduced body fat without affecting lean mass. The results of this study reflect the functional attributes of RoseFit in sustainable weight management.

### Electronic supplementary material

Below is the link to the electronic supplementary material.

Supplementary Material 1

Supplementary Material 2

Supplementary Material 3

Supplementary Material 4

## Data Availability

The datasets used and/or analysed during the current study are available from the corresponding author on reasonable request.

## Abbreviations

ALP: Alkaline phosphatase
ALT: Alanine aminotransferase
AST: Aspartate aminotransferase
BMI: Body Mass Index
CTRI: Clinical Trial Registry
DEXA: Dual Energy X-ray Absorptiometry
ELISA: Enzyme-linked immunosorbent assay
HDL: High density lipoprotein
HFD: High fat diet
HRQL: Health-related quality of life
LDL: Low density lipoprotein
QoL: Quality of Life
RCT: Randomized Clinical Trial
TC: Total cholesterol
TG: Triglycerides
VAS: Visual Analog Scales
