# Supplementary material for: A polyphenol fraction from Rosa multiflora var. platyphylala reduces body fat in overweight humans through appetite suppression – a randomized, double-blind, placebo-controlled trial
Source: BMC Complement Med Ther. 2024 May 21;24:197. doi: 10.1186/s12906-024-04487-1 (PMC11110278; doi:10.1186/s12906-024-04487-1)
Supplement: Supplementary file 1 — Supplementary Material 1 [file 12906_2024_4487_MOESM1_ESM.pdf]

Sample Size Determination

The sample size calculation was based on difference of two treatments are considered to be medically relevant. Assuming a common SD of 2.78 at the end of treatment, 31 per group would be sufficient to detect a difference of 1.45 in mean difference b/w the two treatment with power of 80% and a 0.05. 2-sided level of significance.

```
R-Program:

pwr.t.test( d = 1.45/2.78,   power = 0.8,     type = "paired", alternative
= "two.sided")

     Paired t test power calculation

              n = 30.82683=~31

              d = 0.5215827

      sig.level = 0.05

          power = 0.8

    alternative = two.sided

NOTE: n is number of *pairs*
```

A total of N number of subjects are required at each treatment group in the end of the study with all the data being complete for analysis, but a proportion (q) are expected to drop out before the study ends. In this case, the following total number of subjects ($N1$) would have to be enrolled to ensure that the final sample size (N) in each treatment group is:

$$N1 = \frac{N}{1-q} = \frac{31}{(1-0.10)} = 35$$

Where q is the proportion of attrition and is generally 10% in this type of studies.

Note: The proportion of eligible subjects who will refuse to participate (drop out) or provide the inadequate information will be unknown at the beginning of the study. Approximate estimates is often possible using information from similar studies.
