# Supplementary material for: A polyphenol fraction from Rosa multiflora var. platyphylala reduces body fat in overweight humans through appetite suppression – a randomized, double-blind, placebo-controlled trial
Source: BMC Complement Med Ther. 2024 May 21;24:197. doi: 10.1186/s12906-024-04487-1 (PMC11110278; doi:10.1186/s12906-024-04487-1)
Supplement: Supplementary file 2 — Supplementary Material 2 [file 12906_2024_4487_MOESM2_ESM.pdf]

Table 1. Caloric information on regular diet

Food item	Quantity	Calories
**Breakfast**		
Idli	100 g	338
Sambhar	200 g	
Upma	20 g	480
Chutney	200 g	
Mix Veg Pulav	130 g	760
Bundhi Raita	60 g	
Chapathi	4 no.	
Channa masala	150 g	
Dosa	3 no.	452
Veg sagu	200 g	
**Lunch**		
Chapathi	2 no.	230
Vegetable curry	100 g	
Rice	150 g	
Sambhar	200 g	
Rice	150 g	1015
Sambhar	200 g	
Sheera	100 g	
Paratha	3 no.	
Tindli veg	100 g	
Chapathi	2 no.	750
Tomato curry	100 g	
Rice	150 g	
Dal	150 g	
Vegetable biryani	200 g	550
**Snacks**		
Fruit salad	300 g	270
Sprout masala	150 g	275
Samosa	2 no.	385
Green chutney	20 g	
**Dinner**		
Chapathi	4 no.	950
Vegetable curry	150 g	
Jeera rice	150 g	
Dal	175 g	
Chapathi	4 no.	1020
Palak curry	150 g	
Ghee rice	150 g	
Vegetable kurma	150 g	
Chapathi	3 no.	830
Channa masala	150 g	
Vegetable pulav	120 g	
Gulab Jamun	1 no.	
Roti	3 no.	825
Brinjal/ladies finger veg	100 g	
Vegetable pulav	150 g	
Bundhi raitha	50 g	
Chapathi	3 no.	950
Cabbage veg	100 g	

Rice	150 g	
Sambhar	150 g	
**Non-vegetarian items**		
1 bowl Chicken Dum Biriyani + 1 Bowl Cucumber salad	-	400
Egg mayonnaise sandwich: 2 pieces	-	265
1 bowl rice +chicken curry	-	750
1 mutton Biryani	-	400
Fish meal	-	450
Fish Biryani	-	350
**Sweets**		
Gulab Jamun 2 Pieces	-	350
Jilebi - 3-4 Pieces	-	490
Kheer - 1 bowl	-	250
Laddu - 2 Pieces	-	400

Note: Normal caloric requirement – 2000-2500 cal.
