# Supplementary material for: A polyphenol fraction from Rosa multiflora var. platyphylala reduces body fat in overweight humans through appetite suppression – a randomized, double-blind, placebo-controlled trial
Source: BMC Complement Med Ther. 2024 May 21;24:197. doi: 10.1186/s12906-024-04487-1 (PMC11110278; doi:10.1186/s12906-024-04487-1)
Supplement: Supplementary file 3 — Supplementary Material 3 [file 12906_2024_4487_MOESM3_ESM.pdf]

Per-protocol analysis of efficacy parameters

Table 1. Baseline demographic characteristics of subjects

Variable	RoseFit (n = 28)	Placebo (n = 28)	*p*-value
Age	33.86±7.81	33.71±7.09	0.943[1]
Gender (M/F)	7/21	12/16	0.158[2]

Values are mean ± SD, and data were statistically analyzed using[1] independent sample t-test and [2] Chi-square test. M/F; Male/Female.

Table 2. Effect of RoseFit on body weight and BMI of subjects

**Parameter**	**RoseFit** (N=28)	**Placebo** (N=28)	*p*-value (between groups)
**Body weight (kg)**			
Baseline	72.33±10.69	68.02±11.82	0.158†
Visit 4 (12 weeks)	70.83±10.19	70.88±10.16	0.985†
Change	-1.50±2.86	2.86±5.49	<0.001†***
p-value (Baseline vs Visit 4)	<0.009#**	<0.010#**	
**BMI (kg/m^2)**			
Baseline	28.18±1.53	27.86±1.56	0.528†
Visit 4 (12 weeks)	26.80±1.54	28.65±1.48	<0.001†***
Change	-1.38±0.86	0.79±0.74	<0.001†***
p-value (Baseline vs Visit 4)	<0.001#***	<0.001#***	

Values are presented as mean ± SD. Change = Visit 4 – Baseline
#Paired t-test; †Independent t-test; ***p*<0.01, ****p*<0.001.

Table 3. Effect of RoseFit on the body composition of subjects (DEXA analysis)

**Parameter**	**RoseFit** (N=28)	**Placebo** (N=28)	*p*-value (between groups)
**Body fat (%)**			
Baseline	45.88±7.06	47.87±8.43	0.343†
Visit 4 (12 weeks)	43.77±7.84	49.07±8.61	0.019†*
Change	-2.11±2.74	1.21±3.56	<0.001†***
p-value (Baseline vs Visit 4)	<0.001#***	0.084#	
**Fat mass (kg)**			
Baseline	31.97±5.89	31.57±8.41	0.839†
Visit 4 (12 weeks)	29.78±6.29	33.59±7.57	<0.046†*
Change	-2.19±1.75	2.02±4.19	<0.001†***
p-value (Baseline vs Visit 4)	<0.001#***	0.017#*	
**Lean mass (kg)**			
Baseline	37.96±8.23	34.04±7.50	0.068†
Visit 4 (12 weeks)	38.66±7.89	34.89±7.65	0.075†
Change	0.69±2.25	0.85±2.44	0.805†
p-value (Baseline vs Visit 4)	0.116#	0.077#	

Values are presented as mean ± SD. Change = Visit 4 – Baseline
#Paired t-test; †Independent t-test; *p<0.05, ***p<0.001.

Table 5. Summary of perceived hunger and satiety scores using Visual Analog Scale (VAS)

**Parameter**	**RoseFit** (N=28)	**Placebo** (N=28)	***p*-value (between groups)**
**Hunger score**			
Baseline	88.93±7.37	89.64±8.38	0.883†
Visit 4 (12 weeks)	71.07±13.43	88.21±9.83	<0.001†***
Change	-17.86±13.71	-1.43±8.03	<0.001†***
p-value (Baseline vs Visit 4)	<0.001#***	0.355#	
**Satiety score**			
Baseline	78.21±9.05	80.00±13.61	0.566†
Visit 4 (12 weeks)	86.43±5.59	77.86±10.67	<0.001†***
Change	8.21±9.83	-2.14±7.38	<0.001†***
p-value (Baseline vs Visit 4)	<0.001#***	0.136#	
**Fullness score**			
Baseline	73.57±8.26	73.93±9.94	0.890†
Visit 4 (12 weeks)	83.57±7.31	75.36±6.93	<0.001†***
Change	10.00±9.43	1.43±7.05	<0.001†***
p-value (Baseline vs Visit 4)	<0.001#***	0.293#	
**Prospective food consumption**			
Baseline	86.07±7.86	87.14±11.17	0.680†
Visit 4 (12 weeks)	76.43±6.22	84.29±6.90	<0.001†***
Change	-9.64±9.22	-2.86±10.13	0.011†*
p-value (Baseline vs Visit 4)	<0.001#***	0.147#	

Values are presented as mean ± SD. Change = Visit 4 – Baseline
#Paired t-test (baseline vs. visit 4); †Independent t-test; ***p*<0.01, ****p*<0.001.

Table 6. Effect of RoseFit on serum biomarkers of appetite and satiety

**Parameter**	**RoseFit** (N=28)	**Placebo** (N=28)	*p*-value (between groups)
**Leptin (ng/mL)**			
Baseline	29.80±9.65	34.00±8.17	0.085†
Visit 4 (12 weeks)	28.45±9.46	34.92±8.82	0.011†**
Change	-1.36±1.99	0.92±1.89	<0.001†***
p-value (Baseline vs Visit 4)	0.001#**	0.015#*	
**Ghrelin (pg/dL)**			
Baseline	219.27±26.43	224.23±19.86	0.431†
Visit 4 (12 weeks)	213.04±26.98	224.23±25.79	0.081†
Change	-6.23±5.47	0.00±6.24	<0.001†***
p-value (Baseline vs Visit 4)	<0.001#***	1.000#	

Values are presented as mean ± SD. Change = Visit 4 – Baseline
#Paired t-test (baseline vs. visit 4); †Independent t-test; ***p*<0.01, ****p*<0.001.
