# Supplementary material for: A polyphenol fraction from Rosa multiflora var. platyphylala reduces body fat in overweight humans through appetite suppression – a randomized, double-blind, placebo-controlled trial
Source: BMC Complement Med Ther. 2024 May 21;24:197. doi: 10.1186/s12906-024-04487-1 (PMC11110278; doi:10.1186/s12906-024-04487-1)
Supplement: Supplementary file 4 — Supplementary Material 4 [file 12906_2024_4487_MOESM4_ESM.pdf]

Adverse events reported during the study

Table 1. Summary of adverse events (Safety population)

Adverse Events	GROUP A (RoseFit) (N=35) n (%)	GROUP B (Placebo) (N=35) n (%)	Overall (N=70) n (%)
Total Number of AEs Reported	06	09	15
Subjects Reporting at least one AEs	03 (8.6%)	04 (11.4%)	07 (10%)
Total Number of SAEs Reported	00	00	00
Subjects Reporting Serious AEs	00	00	00
Subjects Reporting drug-related AEs	00	00	00
Subjects Reporting AEs leading to early discontinuation	00	02 (5.7%)	02 (2.8%)
Number of Deaths	00	00	00

Table 2. Details of AEs experienced by the subjects (Safety population)

Adverse Events Term	GROUP A (RoseFit) (N=35) n (%)	GROUP B (Placebo) (N=35) n (%)	Overall (N=70) n (%)
Subjects with atleast one AE	03 (8.6%)	04 (11.4%)	07 (10%)
Headache	03 (8.6)	01 (2.8)	04 (5.7)
Stomach ache	00	02 (5.7)	02(2.8)
Viral fever	02 (5.7)	00	02 (2.8)
Cold	01 (2.8)	01(2.8)	02 (2.8)
Sore throat	00	01 (2.8)	01 (1.4)
Fever	00	01 (2.8)	01 (1.4)
Bloating	00	01 (2.8)	01 (1.4)
Heart burn	00	01 (2.8)	01 (1.4)
Body Ache	00	01 (2.8)	01 (1.4)
